# Trait modality distribution of aquatic macrofauna communities as explained by pesticides and water chemistry

**DOI:** 10.1007/s10646-016-1671-5

**Published:** 2016-05-21

**Authors:** O. Ieromina, C. J. M. Musters, P. M. Bodegom, W. J. G. M. Peijnenburg, M. G. Vijver

**Affiliations:** Institute of Environmental Sciences, Leiden University, P.O. Box 9518, 2300 Leiden, RA The Netherlands; National Institute for Public Health and Environment, P.O. Box 1, 3720 Bilthoven, BA The Netherlands

**Keywords:** Aquatic community, Traits, Pesticides stress, Environmental factors, Nutrients

## Abstract

Analyzing functional species’ characteristics (species traits) that represent physiological, life history and morphological characteristics of species help understanding the impacts of various stressors on aquatic communities at field conditions. This research aimed to study the combined effects of pesticides and other environmental factors (temperature, dissolved oxygen, dissolved organic carbon, floating macrophytes cover, phosphate, nitrite, and nitrate) on the trait modality distribution of aquatic macrofauna communities. To this purpose, a field inventory was performed in a flower bulb growing area of the Netherlands with significant variation in pesticides pressures. Macrofauna community composition, water chemistry parameters and pesticide concentrations in ditches next to flower bulb fields were determined. Trait modalities of nine traits (feeding mode, respiration mode, locomotion type, resistance form, reproduction mode, life stage, voltinism, saprobity, maximum body size) likely to indicate pesticides impacts were analyzed. According to a redundancy analysis, phosphate -and not pesticides- constituted the main factor structuring the trait modality distribution of aquatic macrofauna. The functional composition could be ascribed for 2–4 % to pesticides, and for 3–11 % to phosphate. The lack of trait responses to pesticides may indicate that species may have used alternative strategies to adapt to ambient pesticides stress. Biomass of animals exhibiting trait modalities related to feeding by predation and grazing, presence of diapause form or dormancy, reproduction by free clutches and ovoviviparity, life stage of larvae and pupa, was negatively correlated to the concentration of phosphate. Hence, despite the high pesticide pollution in the area, variation in nutrient-related stressors seems to be the dominant driver of the functional composition of aquatic macrofauna assembly in agricultural ditches.

**Electronic supplementary material:**

The online version of this article (doi:10.1007/s10646-016-1671-5) contains supplementary material, which is available to authorized users.

## Introduction

Traditionally, the responses of biotic communities to human-induced disturbances have been evaluated based on taxonomic approaches, e.g. by estimating the species composition or the performance of selected indicator species (Mouillot et al. 2006). During the recent decades, the use of traits-based approaches, i.e. characterizing communities according to functional characteristics, has gained an increasing interest. The reason is that functional traits were shown to reflect the mechanisms underlying community responses to environmental drivers (Poff 1997; Statzner and Beche 2010). Information obtained using traits-based approaches may thus be extrapolated to a broader range of species and geographical zones (Baird et al. 2008; Dolédec et al. 2006; Charvet et al. 2000). These approaches have been successfully developed for a wide array of plant (for instance, Engelhardt 2006; Quétier et al. 2007; Suding et al. 2008; Van Bodegom et al. 2014) and animal communities, including invertebrates (for instance, Culp et al. 2011; Poff et al. 2006; Charvet et al. 2000; Dolédec et al. 2006; Vieira et al. 2006; Magbauna et al. 2010; Menezes et al. 2010; Statzner and Beche 2010; Ippolito et al. 2012). Increasingly, traits-based approach is also applied to understand the impacts of pesticides on community responses of aquatic invertebrate communities (Liess and Von Der Ohe 2005; Ippolito et al. 2012; Rubach et al. 2010).

So far, traits-based approaches characterizing invertebrate community responses to pesticides have mostly treated pesticides impacts in isolation. However, in multi-stressor field conditions, pesticides are not the only drivers of invertebrate community composition. A number of key environmental factors varying over time and space may influence the performance of aquatic biota in water systems around agricultural areas. First, the use of pesticides in the agricultural fields results in the presence of pesticide mixtures in surface waters. Therefore, aquatic biota may be affected by mixtures of pesticides. Second, nutrients (phosphorus and nitrogen) are commonly applied to the fields to enhance yields and are often transported to surface waters in relatively large amounts along with pesticides (EPA 2012; Tilman et al. 2002). Nutrients were shown to affect the responses of aquatic invertebrates to pesticides in the laboratory and semi-field conditions (Alexander et al. 2013; Ieromina et al. 2014a, b). Third, other physico-chemical parameters also vary strongly across surface waters around agricultural fields. Several semi-field studies have considered factors other than pesticides in evaluating the effects of pesticides on the community trait composition (for instance, Knillmann et al. 2012; Liess and Beketov 2011; Liess et al. 2008, 2013). However, to which extent these drivers affect the trait composition, i.e., the distribution of the modalities per trait within a community, in a field situation remains poorly understood.

This study therefore aimed (1) to analyze the relationships between trait modality distributions of aquatic macrofauna for a range of traits, pesticides and environmental factors; and (2) to quantify the contribution of individual pesticides and environmental factors to the total variance in the trait modality distribution. Traits likely to respond to chemical stress, such as traits related to the external exposure (feeding mode, life stage), internal sensitivity (respiration mode, maximum body size), population recovery (locomotion type, resistance form, voltinism, reproduction mode) (as classified by Rubach et al. 2011), and sensitivity to pollution as reflected in physiological and biochemical characteristics (saprobity) were analyzed. We hypothesized that (1) both pesticides and environmental factors will significantly affect the trait modality distribution of aquatic macrofauna; and (2) the contribution of pesticides to the total variance in the trait modality distribution per trait will be comparable to that of other environmental factors that vary considerably in agricultural areas, and are inherently important for aquatic biota.

## Materials and methods

### Macrofauna sampling, measurements of environmental parameters and pesticide concentrations

A total of 18 sites in a freshwater ditch system located in the flower bulb growing region of the Netherlands were sampled repeatedly four times in the period April–November 2011–2012 with a time interval of 1–1.5 months: 10 sites located in ditches next to flower bulb fields, 4 ditches next to pastures, and 4 sites located in watersheds of nature reserve close to the flower bulb area. The map of the study area can be found in Fig. S1. The depth of the ditches was minimally 0.7–1 m, and selected ditches do not dry during the year. A detailed description of the research area, macrofauna sampling strategy and taxonomic identification level for each group is given in Ieromina et al. (2016). In brief, macrofauna samples were collected using a dipping net dragged over the total length of 5 m using a multihabitat sampling strategy. Afterwards, macrofauna samples were brought to the lab and identified to the lowest taxonomic level feasible, hereafter called ‘taxon’. The following water chemistry parameters were monitored: temperature (T, °C), dissolved oxygen (DO, mg/L), pH, conductivity (mS), dissolved organic carbon (DOC, mg/L). Floating macrophytes cover (Macr) was estimated in order to account for habitat structure. Measurements of the concentrations of phosphate (PO_4_^3−^), nitrite (NO_2_^−^), nitrate (NO_3_^−^) and pesticides commonly applied in bulb fields (chlorprofam, pirimiphos-methyl, tolclophos-methyl, carbendazim, ethiofencarb, imidacloprid, isoproturon, imazalil, methiocarb, and prochloraz) were performed in the OMEGAM laboratory (Amsterdam, the Netherlands) using standard protocols. An overview of the main characteristics of water chemistry and pesticide concentrations at the sampling sites is given in Supplementary Info (Tables S1 and S2), an overview of the biotic data underlying the analyses is given in Supplementary Information Table S3. From Table S2, it can be concluded that pesticide concentrations were found above the limit of detection at most of the locations, and therefore were high enough to expect an induction of effects.

### Assigning trait modalities

Each macrofauna taxon was classified into pre-defined trait modalities of nine traits: feeding mode, locomotion type, resistance form, voltinism, reproduction mode, life stage, respiration mode, body size and saprobity (Table 1). Trait data were retrieved from the online database www.freshwaterecology.info (Schmidt-Kloiber and Hering 2012 accessed in the years 2012–2014, last accessed 04.04.2014) supplemented by literature available through the Web of Science (http://apps.webofknowledge.com/). If a taxon was characterized by more than one modality of a trait, each of these modalities was assigned a coefficient ranging from 0 to 1, depending on how abundantly the given modality is represented in this taxon. For instance, the trait “feeding mode” included 7 modalities: deposit feeding, predating, grazing, shredding, filter feeding, gathering and parasite type of feeding. If a taxon feeds 80 % by grazing and 20 % by predation, then the modality “grazing” was assigned a coefficient 0.8 and modality “predation” was assigned a coefficient 0.2. If a taxon was characterized by one modality of a trait, this modality was assigned a coefficient of 1, and the other modalities of this trait were assigned 0. As a result, a species—trait modality matrix was obtained for each trait.

**Table 1:** List of traits and trait modalities used to classify macrofauna taxa

Trait category	Trait*	Trait modality	Abbreviation
Physiological	Feeding mode	Deposit feeders	FDep
		Predators	FPred
		Grazers	FGraz
		Shredders	FShred
		Filter feeders	FFIlt
		Gatherers and/or collectors	FGath
		Parasites	FPar
	Respiration mode	Gill respiration	RGill
		Aerial respiration (hydrostatic vesicle)	RAir
		Plastron	RPlas
		Tegument respiration	RTeg
Dispersal	Locomotion type	Scatting	LScat
		Diving	LDiv
		Sprawling, walking	LWalk
		Sessile	LSess
		Burrowing	LBur
	Resistance form	Egg and/or statoblast	ResEgg
		Cocoons	ResCoc
		Houses against desiccation	ResHous
		Diapause and/or dormancy	ResDiap
		Quiescence	ResQui
		None	ResNone
Life history	Reproduction type	Ovoviviparity	ROviv
		Free isolated eggs	RFreeE
		Fixed clutches	RFixCl
		Free clutches	RFreeCl
		Clutches in vegetation	RClVeg
	Life stage	Pupa	Pupa
		Larvae	Larv
		Adult	Ad
	Voltinism	Semivoltine	Sev
		Bivoltine	Biv
		Multivoltine	Mult
		Univoltine	Uni
		Trivoltine	Triv
		Flexible	Flex
Ecological	Saprobity	Xenosaprob	Xeno
		Oligosaprob	Oligo
		Beta-mesosaprob	Beta
		Alpha-mesosaprob	Alpha
		Polysaprob	Poly
Morphological	Maximum body size	0.05–1 cm	0.05–1
		1 cm–2 cm	1–2
		2 cm–5 cm	2–5
		5 cm–10 cm	5–10

* Traits and trait modalities were selected based on literature data: Rubach et al. (2011); Magbauna et al. (2010); Statzner and Beche (2010); Vieira et al. (2006); Ippolito et al. (2012); Charvet et al. (2000)

To express the community trait modality distribution at each site-time combination, trait observations for each taxon within the community were weighted by their abundance (number of individuals) and the individual biomass. For this purpose, data on the maximum body size for each taxon were collected from literature and added to the trait modality matrix. After that, the trait modality coefficient of each taxon was multiplied by the body size, and by the abundance of the given taxon within the sample. This weighing avoids unduly impacts of small rare species on community trait expressions and concurs to the biomass ratio hypothesis (Grime 1998).

### Statistical analysis

The trait modality distribution as affected by environmental factors and pesticides was analyzed by redundancy analysis (RDA) for each trait separately. Community trait modalities were included in the analyses as response variables, while the concentrations of individual pesticides (chlorprofam, pirimiphos-methyl, tolclophos-methyl, carbendazim, ethiofencarb, imidacloprid, isoproturon, imazalil, methiocarb, and prochloraz) and environmental factors [temperature, dissolved oxygen (DO), DOC, nitrate, nitrite, phosphate, macrophyte cover] were explanatory variables. To account for macrofauna ontogeny, the number of the month of the year was included in the analysis as a nominal covariate. By accounting for season as a variable, the impacts of other variables, otherwise potentially confounded by season, could be determined in an unbiased fashion. The number of explanatory variables (17) was lower than the number of site-time combinations (79) fulfilling the requirements of a RDA.

Prior to the RDA, the skewness (the symmetry of distribution), the kurtosis (the shape of the distribution), and the normality of the distribution (Shapiro–Wilk test) were tested for each variable. To increase the normality and reduce skewness, and following recommendations of Legendre and Birks (2012), data were log (x + 1) transformed. For many parameters, skewness were within the range between −2 and 2, which corresponds to a univariate normal distribution (George and Mallery 2010). In addition, prior to RDA, data were centered and standardized by error variance. The significance of all canonical axes per trait (which represents whether the explanatory variables explain a significant part of the variation in trait modality) and of the first RDA axis (showing whether a significant part of this explained variance is displayed on the first axis) were tested by the Monte Carlo permutation test (based on 999 unrestricted permutations), and Eigenvalues, F-ratios and *p* values were derived.

The total variance explained by individual pesticides and environmental factors was calculated based on the sum of all canonical eigenvalues, and was expressed as a percentage relative to the total variance. To assess collinearity between explanatory variables, the variance inflation factor (VIF) was calculated for each explanatory variable. VIF reflects the amount of variance in regression coefficient increased as a result of collinearity between explanatory variables (Verspoor et al. 2011). The contribution of each individual explanatory variable to the total explained variance was quantified using the Monte Carlo permutation test following an automated forward selection procedure (based on 499 permutations). Normality tests were performed in SPSS software (Version 21, IBM Corp. Released 2012). Multivariate analysis was performed in CANOCO software v.4.5 (Braak and Šmilauer 2002).

## Results

### Linking trait modalities, pesticides and environmental factors

The results of the Monte Carlo permutation test indicated that the first ordination axis was significant for the traits resistance form, feeding mode, reproduction type, and aquatic life stage, meaning that there was a significant relationship between the trait modality distributions of these traits, pesticides and environmental factors (Table 2). The trait modalities of the other traits (respiration mode, voltinism, saprobity, maximum body size, locomotion type) were not significantly correlated to explanatory variables included in the RDA.

**Table 2:** Summary of Monte Carlo test (based on 999 permutations) identifying the significance of the first canonical axis and the significance of all canonical axes in RDA presented in Figs. 1 and 2

		Test of significance of first canonical axis	Test of significance of all canonical axes
Resistance form			
Eigenvalue		0.106	0.209
F-ratio		8.5	1.3
*p* value		0.026**	0.11
Reproduction type			
Eigenvalue		0.115	0.168
F-ratio		10.0	1.0
*p* value		0.074*	0.396
Saprobity			
Eigenvalue		0.157	0.223
F-ratio		12.6	1.3
*p* value		0.108	0.152
Respiration			
Eigenvalue		0.11	0.181
F-ratio		8.8	1.1
*p* value		0.13	0.39
Feeding mode			
Eigenvalue		0.154	0.263
F-ratio		11.9	1.6
*p* value		0.006**	0.032**
Voltinism			
Eigenvalue		0.086	0.191
F-ratio		6.4	1.1
*p* value		0.162	0.308
Aquatic life stage			
Eigenvalue		0.179	0.247
F-ratio		14.1	1.4
*p* value		0.036**	0.09*
Locomotion type			
Eigenvalue		0.124	0.201
F-ratio		9.4	1.1
*p* value		0.142	0.31
Body size			
Eigenvalue		0.073	0.198
F-ratio		5.1	1.1
*p* value		0.672	0.332

* *p* < 0.1; ** *p* < 0.05

**Fig. 1:**
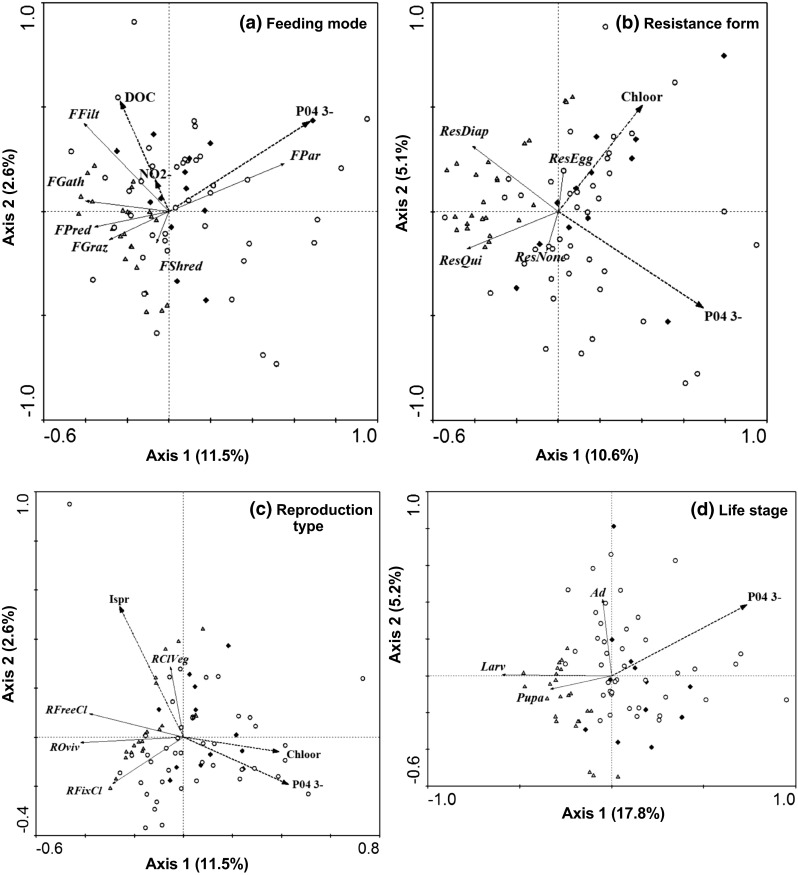
Redundancy analysis plot of macrofauna trait modality distribution per trait weighted by biomass (feeding mode (**a**), resistance form (**b**), reproduction type (**c**), aquatic life stage (**d**)), pesticides and environmental factors. Shown in the graph are traits for which the first RDA ordination axis was significant (*p* < 0.1). Abbreviations for the trait modalities can be found in Table 1. *Dashed line* pesticides and environmental factors, *solid line* trait modalities. *Chloor* chlorpropham. *Triangular* sites in watersheds of nature reserve, *circles* sites in ditches next to flower bulb fields, *diamonds* sites in ditches next to pastures. *Chloor* chlorpropham, *Ispr* isoproturon. Only the explanatory variables explaining a significant part of variance in trait modality distribution are shown

Overall, the total explained variance in the trait modality distribution per trait varied from 16.8 % for trait reproduction type to 26.3 % for trait feeding mode. Of all variables included, phosphate tended to contribute most to explaining the variance in the trait modality distribution per trait, as identified by the Monte Carlo permutation test (the contribution of phosphate to the total variance varied between 3 and 11 %) (Table 3), while the contribution of pesticides to the total explained variance varied between 2 and 4 %. Phosphate was positively correlated to the biomass of parasites (Fig. 1a), and negatively to the biomass of animals exhibiting the following traits: feeding by predation and grazing (Fig. 1a), presence of diapause form or dormancy (Fig. 1b), reproduction by free clutches and ovoviviparity (that was also negatively correlated to chlorpropham) (Fig. 1c), life stage of larvae and pupa (Fig. 1d).

**Table 3:** Summary statistics of redundancy analysis (RDA) of taxon traits weighted by biomass (trait modalities per trait were inlcuded in the response variable dataset), pesticides and environmental factors (explanatory variable dataset): sum of all canonical eigenvalues (Sum λ), individual explanatory variables identified to be statistically significant according to Monte Carlo permutation test (based on 499 permutations), variance explained by each individual explanatory variable, F test, and *p* value

Response variable	Sum λ	Explanatory variables	Variance explained	F	*p* value
Resistance form	0.209	P0_4_^3−^	0.07	6.53	0.002**
		Chloor	0.04	3.51	0.008**
Reproduction type	0.168	Chloor	0.03	2.45	0.068*
		Ispr	0.02	2.64	0.08*
		P0_4_^3−^	0.03	2.33	0.068*
Feeding mode	0.263	PO_4_^3−^	0.08	7.03	0.002**
		NO_2_^−^	0.04	3.13	0.008**
		DOC	0.04	3.38	0.01**
Aquatic life stage	0.247	PO_4_^3−^	0.11	9.46	0.002**

Shown in the table are traits for which the first RDA ordination axis was significant (*p* < 0.1).Only explanatory variables identified to be statistically significant (*p* < 0.1) are represented

* *p* < 0.1; ** *p* < 0.05

In addition, positive correlations were found between the biomass of filter-feeders, DOC and nitrite concentration (Fig. 1a); that of animals having resistance form of egg or statoblast and the concentration of chlorpropham (that was also negatively correlated to animals with no resistance form or resistance form of quiescence) (Fig. 1b); and that of animals reproducing by clutches in vegetation and the concentration of isoproturon (Fig. 1c).

**Fig. 2:**
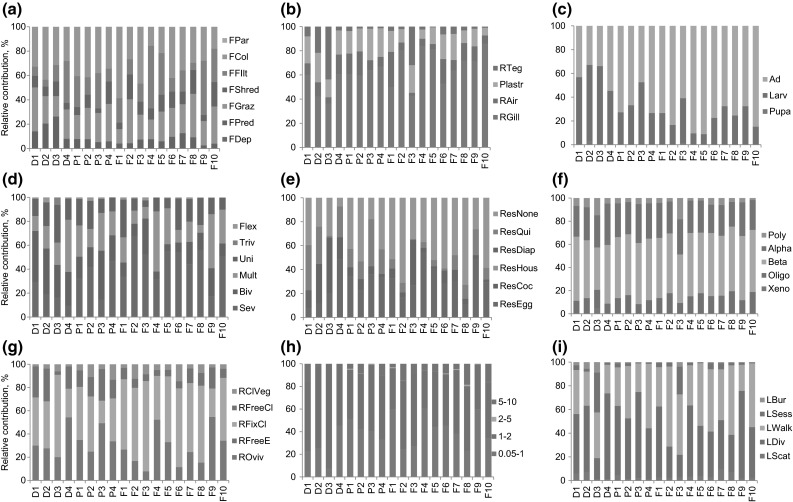
Relative occurrences of trait modalities per trait for each study site (*D* sites in watersheds of the nature reserve, *P* sites in ditches next to pastures; *F* sites in ditches next to flower bulb fields). Shown are the mean values for study site. *a* feeding mode, *b* respiration mode, *c* life stage, *d* voltinism, *e* resistance form, *f* saprobity, *g* reproduction type, *h* maximum body size, *i* locomotion type. Explanations of the abbreviations for the trait modalities can be found in Table 1

A high biomass of animals having diapause form or dormancy, feeding by predation and found at larvae or pupa life stages was found at the nature reserve sites (Fig. 2). Biomass of animals reproducing by clutches in vegetation, and feeding by parasitism was higher in agricultural ditches.

## Discussion

### Relative importance of pesticides and environmental factors in structuring trait modality distribution

Overall, the total explained variance in trait modality distribution per trait did not exceed 26 % (Fig. 1). Also other field studies found that only a small proportion of variance in aquatic community composition (20–30 %) could be explained by field-relevant factors (Larsen et al. 2012; Zuellig et al. 2012). While is seems unlikely that all field studies failed to account for the majority of the environmental drivers, these results suggest that macroinvertebrates have a large array of alternative strategies to deal with the environmental pressures of aquatic systems.

Pesticides were hardly related to trait modalities, while phosphate (and not pesticides, carbon sources, oxygen, pH, temperature, or other nutrients, such as nitrate or nitrite) contributed most to explaining the functional composition of aquatic macrofauna assembly in agricultural ditches. This conclusion is based on results of RDA showing that phosphate contributed significantly to the variance in trait modality distribution of all traits for which significant correlations with explanatory variables were found (Fig. 1). At most of the study sites located within the agricultural area, the concentration of phosphate greatly exceeded the water quality standard of 1 mg/L—the concentration above this threshold reflects deteriorating water quality (according to UKTAG 2012), while pesticides concentrations in the research area remained largely below toxicity thresholds (Ieromina et al. 2014a, b). This suggests that due to its relatively high concentration, the effects of phosphate have possibly prevailed over the effects of pesticides. Phosphorus represents one of the key elements in the aquatic biogeochemical cycle. Being an essential nutrient, phosphorus influences the phytoplankton primary production in aquatic ecosystems, and limits the performance of benthic and planktonic invertebrates. As found in the study of Scheffer et al. (2002), nutrient enrichment in freshwater ecosystems (independent of its type) causes shifts in the structure of aquatic vegetation towards the dominance by phytoplankton. This results in the increase of water turbidity leading to reduced dissolved oxygen concentrations, shading and low light availability for aquatic biota. Therefore, nutrient enrichment may induce a cascade of direct and indirect effects in aquatic ecosystems. Application of fertilizers (nitrogen and phosphors) is a common practice in the research area (flower bulb growing region of the Netherlands). Fertilizers in the area are applied in relatively high amounts (Centraal Bureau voor de Statistiek 2015), which can explain relatively high concentrations of nutrients found in surface waters. Continuous nutrient enrichment in surface waters combined with inherent importance of phosphate to freshwater ecosystems can possibly explain high contribution of this nutrient to the variance in the trait modality distribution of aquatic macrofauna. Trait modalities of traits related to resistance form, respiration, reproduction and life stage (i.e. all traits found to be significantly associated pesticides and environmental factors) were affected by phosphate.

In contrast, pesticides did not appear to be an important factor structuring aquatic macrofauna. Species may have multiple alternative strategies to adapt to pesticide stress. The use of alternative strategies can lead to the shifts in trait modality distributions of many traits. However, in our results such shifts in response to pesticides were not evident. Only herbicides chlorphrofam and isoproturon did affect the trait modality distribution of traits resistance form and reproduction type. This high relative importance of herbicides compared to other categories of pesticides can possibly be explained by their mode of action targeted at the suppression of plant growth. The transfer of herbicides from the agricultural fields to the ditches can lead to side effects of herbicides to aquatic plants and algae that constitute a major food source for many macrofaunal organisms. In addition, aquatic plants impact invertebrates indirectly through changing oxygen levels in water, and providing shelter. Therefore there are various indirect processes can possibly explain the observed correlation between herbicides and invertebrates.

Alternatively, macrofauna community have possibly adapted to pesticide stress without affecting the trait modality distribution per trait. The underlying mechanism for this explanation is that if one species disappears, another species characterized by a similar combination of traits can replace it and thus ecosystem functions are maintained (Cleland 2011). Such compensation mechanism may take place when the extent of disturbance is relatively low, so that it does not induce a pressure on a community assembly (which would coincide with the low pesticide concentrations).

While it is difficult to distinguish causes and effects in field studies due to potentially confounding factors, in this particular system the RDA showed that pesticide concentrations generally varied independently from nutrients (Fig. 1). In addition, the variance inflation factor (VIF) for all explanatory variables was maximally four (Supplemental Info, Table S4), which means that collinearity between explanatory variables was not substantial (O’Brien 2007) and high values did not include combinations of pesticides and nutrients. Thus, in this field situation, such confusion of effects seems to be highly unlikely.

Seasonality may also affect macrofauna community composition and abundances (Šporka et al. 2006). According to Van den Brink et al. (In press 2015), different nymphs species of the mayflies from the overwintering generation react significantly differently to neonicotinoid pollution than those from the summer generations. In our work, the observations in the field had been done over a time span of 7 months each year. By including the number of the month as a co-variate, and by evaluating the coincidence of pesticides and nutrients with ‘month’, we ensured to include such potential seasonal effects. Therefore, the effect of seasonality was accounted for in our analysis and did not influence our results.

### Relationships between trait modalities, pesticides and environmental factors

The importance of including species traits in biomonitoring studies was highlighted in previous studies (Culp et al. 2011). According to our results, the trait modality distribution of traits related to resistance form, respiration, reproduction and life stage were significantly affected by pesticides and environmental factors.

Predators were mainly associated with clean waters of the nature reserve. It is well known that chemicals accumulate through the food web (Ellgehausen et al. 1980). Predators represent the upper level of the food chain, and are exposed to higher concentrations of chemicals, compared to organisms of the lower trophic levels. Being exposed through both habitat and food, predators tend to take up high amounts of chemicals, also when the exposure through the habitat is low (Rubach et al. 2011). This possibly resulted in a high sensitivity of predators to nutrients, as observed in our study. Filter-feeders, feeding on suspended fine and course particulate organic matter (FPOM and CPOM) (Schmidt-Kloiber and Hering 2012), were positively correlated to DOC. This indicates that high DOC concentration in water, possibly associated with high FPOM and CPOM content, favored filter-feeders.

The resistance form of egg or statoblast was positively correlated to chlorprofam. The presence of such resistance form possibly helped the organisms to withstand the impact of pesticide stress, in contrast to organisms without any resistance form. The presence of diapause form or dormancy is commonly described as a feature of the disturbed environment (Díaz et al. 2008). However, this result was not confirmed by our study. Instead, this trait was more likely to be found in the nature reserve.

The production of isolated eggs and ovoviviparity (type of reproduction in which eggs stay inside the body until hatching) (Schmidt-Kloiber and Hering 2012) were characteristic of clean waters. Similarly, Díaz et al. (2008) found that reproduction by isolated eggs is a feature of an undisturbed environment. The authors suggested that the total surface area of single isolated eggs is higher than the surface area of the egg clutch, what makes isolated eggs more sensitive to chemicals (Díaz et al. 2008) possibly due to the high adsorption of chemicals associated with large surface-volume ratio. As a result, animals reproducing by isolated clutches may have been negatively affected by phosphate and chlorpropham, as seen in our study. Ovoviviparity does not involve parental care and therefore was described to be typical of highly disturbed environments (Dolédec et al. 1999; Díaz et al. 2008) that contrasted with our findings. As a possible explanation, offspring hatched within the body and distributed directly to the water column was sensitive to water quality, and could better survive in clean waters of the nature reserve. Other reproduction types involve production of egg clutches—either free or fixed to vegetation or other substrates. Reproduction by clutches in vegetation was mainly attributed to agricultural ditches. Fixed egg clutches are not easily removed by water flow meaning that animals characterized by this reproduction type can survive in highly dynamic conditions of agricultural ditches. High sensitivity of animals having aquatic life stage was highlighted in the previous studies (Liess and von der Ohe 2005), and was also confirmed by our results.

Hence, contaminated conditions of agricultural ditches possibly induced selective pressure on the macrofauna community assembly favoring species characterized by a combination of traits allowing them to survive and reproduce in a highly disturbed environment. According to our results, such advantageous traits were parasite type of feeding, diapause form of egg or statoblast, and reproduction by clutches in vegetation. Animals not exhibiting these traits most likely were not able to live in the disturbed environment. Traits such as feeding by predation and grazing, presence of diapause form or dormancy, pupa or larvae life stage, reproduction by ovoviviparity and free clutches were more typical of clean waters. Because of their high sensitivity to contaminated conditions of agricultural ditches, these traits can be analyzed in ecological impact assessment practices, as possible indicators of pesticide and nutrient pollution. The insights may also be used to develop new practices for biodiversity conservation that aims at preserving both the taxonomic and functional diversity.

## Conclusions

The results of the traits-based approach showed that the trait modality distribution across ditches next to flower bulb fields and nature reserves was strongly driven by phosphate. These results suggest that agricultural pollution in ditches (mainly related to phosphate) induce selective pressure on the trait composition of the macrofauna. Our results indicate that macrofauna traits related to resistance form, feeding mode, reproduction type, and life stage can potentially be analysed to monitor the ecological status of aquatic ecosystems in ecological assessment practices.

## Electronic supplementary material

Below is the link to the electronic supplementary material.

Supplementary material 1 (DOCX 1137 kb)
