# Supplementary material for: Trait modality distribution of aquatic macrofauna communities as explained by pesticides and water chemistry
Source: Ecotoxicology. 2016 May 21;25:1170–80. doi: 10.1007/s10646-016-1671-5 (PMC4921112; doi:10.1007/s10646-016-1671-5)
Supplement: Supplementary file 1 — Supplementary material 1 (DOCX 1137 kb) [file 10646_2016_1671_MOESM1_ESM.docx]

Table S1. Mean and standard deviation (in italic) of water chemistry parameters measured at the sampling sites (from Ieromina et al, 2015)

| Site | T | pH | Cond | DO | DOC | P | Nitrite | Nitrate |
| --- | --- | --- | --- | --- | --- | --- | --- | --- |
| D1 | 15.25 | 8.07 | 249.92 | 6.17 | 141.23 | 0.03 | 0.01 | 0.05 |
|  | *4.71* | *1.13* | *220.21* | *2.32* | *132.99* | *0.01* | *0.00* | *0.07* |
| D2 | 15.10 | 7.55 | 401.12 | 5.24 | 187.43 | 0.03 | 0.01 | 0.05 |
|  | *5.50* | *0.47* | *297.15* | *2.40* | *204.33* | *0.01* | *0.00* | *0.07* |
| D3 | 15.58 | 7.47 | 622.25 | 6.26 | 63.33 | 0.03 | 0.01 | 0.05 |
|  | *3.38* | *0.52* | *50.49* | *2.50* | *32.32* | *0.01* | *0.00* | *0.07* |
| D4 | 15.05 | 7.69 | 617.50 | 6.40 | 48.27 | 0.03 | 0.01 | 0.05 |
|  | *3.27* | *4.70* | *51.80* | *2.00* | *5.57* | *0.01* | *0.00* | *0.07* |
| P1 | 15.85 | 7.75 | 612.59 | 4.83 | 163.70 | 2.46 | 0.03 | 0.13 |
|  | *5.08* | *0.11* | *306.96* | *1.64* | *128.36* | *2.48* | *0.03* | *0.19* |
| P2 | 14.73 | 7.69 | 726.77 | 5.01 | 181.97 | 0.91 | 0.02 | 0.19 |
|  | *4.35* | *0.48* | *376.44* | *2.21* | *119.18* | *0.44* | *0.01* | *0.24* |
| P3 | 15.78 | 7.56 | 760.75 | 6.37 | 87.27 | N | N | N |
|  | *5.87* | *0.55* | *66.09* | *5.82* | *68.13* |  |  |  |
| P4 | 14.73 | 7.46 | 729.50 | 5.45 | 86.88 | N | N | N |
|  | *5.25* | *0.31* | *92.42* | *2.36* | *60.85* |  |  |  |
| F1 | 14.48 | 7.66 | 547.54 | 4.37 | 215.42 | 1.83 | 0.04 | 0.15 |
|  | *3.57* | *0.31* | *418.78* | *1.67* | *144.03* | *1.38* | *0.01* | *0.08* |
| F2 | 14.77 | 7.68 | 467.32 | 3.71 | 193.96 | 0.78 | 0.02 | 0.06 |
|  | *3.83* | *0.42* | *351.88* | *1.43* | *217.22* | *0.99* | *0.01* | *0.07* |
| F3 | 14.74 | 7.90 | 450.25 | 4.67 | 286.40 | 2.01 | 0.05 | 0.23 |
|  | *5.86* | *0.74* | *634.63* | *2.14* | *112.96* | *1.58* | *0.04* | *0.19* |
| F4 | 15.23 | 8.09 | 607.27 | 4.45 | 290.97 | N | N | N |
|  | *4.73* | *0.62* | *359.19* | *1.91* | *360.86* |  |  |  |
| F5 | 15.67 | 7.98 | 712.42 | 4.76 | 152.58 | 1.12 | 0.04 | 0.18 |
|  | *4.46* | *0.65* | *358.91* | *2.10* | *124.94* | *0.44* | *0.02* | *0.33* |
| F6 | 16.02 | 8.20 | 752.94 | 5.34 | 233.32 | 4.10 | 0.03 | 0.33 |
|  | *4.50* | *0.99* | *403.71* | *1.61* | *144.41* | *3.24* | *0.03* | *0.49* |
| F7 | 15.02 | 7.99 | 816.43 | 5.20 | 173.37 | N | N | N |
|  | *4.29* | *0.67* | *408.01* | *1.92* | *137.78* |  |  |  |
| F8 | 15.35 | 8.11 | 851.24 | 6.10 | 179.27 | 1.96 | 0.07 | 0.60 |
|  | *4.43* | *0.75* | *425.48* | *1.47* | *164.93* | *0.79* | *0.07* | *0.66* |
| F9 | 14.52 | 8.13 | 617.86 | 4.60 | 247.11 | N | N | N |
|  | *5.11* | *0.99* | *536.43* | *2.70* | *126.32* |  |  |  |
| F10 | 15.28 | 7.98 | 743.59 | 5.58 | 153.43 | 1.48 | 0.01 | 0.05 |
|  | *5.09* | *0.69* | *370.61* | *1.72* | *103.93* | *1.32* | *0.01* | *0.04* |

Table S2. Mean and standard deviation (in italic) of pesticide concentrations (µg/L) measured at the sampling sites (from Ieromina et al, 2015)

|  | Chloor | PirM | TolM | Proch | Carb | Ethfc | Imzl | Imdc | Ispr | Meth |
| --- | --- | --- | --- | --- | --- | --- | --- | --- | --- | --- |
| D1 | 0.010 | 0.005 | 0.005 | 0.100 | 0.010 | 0.025 | 0.005 | 0.024 | 0.005 | 0.010 |
|  | *0* | *0* | *0* | *0* | *0* | *0* | *0* | *0* | *0* | *0* |
| D2 | 0.010 | 0.005 | 0.005 | 0.100 | 0.010 | 0.025 | 0.005 | 0.024 | 0.005 | 0.010 |
|  | *0* | *0* | *0* | *0* | *0* | *0* | *0* | *0* | *0* | *0* |
| D3 | 0.010 | 0.005 | 0.005 | 0.100 | 0.010 | 0.025 | 0.005 | 0.024 | 0.005 | 0.010 |
|  | *0* | *0* | *0* | *0.045* | *0* | *0* | *0* | *0* | *0* | *0* |
| D4 | 0.010 | 0.005 | 0.005 | 0.100 | 0.010 | 0.025 | 0.005 | 0.024 | 0.005 | 0.010 |
|  | *0* | *0* | *0* | *0* | *0* | *0* | *0* | *0* | *0* | *0* |
| P1 | 0.030 | 0.035 | 0.005 | 0.100 | 0.650 | 0.025 | 0.005 | 0.046 | 0.005 | 0.010 |
|  | *0.045* | *0.064* | *0* | *0* | *1.259* | *0* | *0* | *0.047* | *0* | *0* |
| P2 | 0.019 | 0.030 | 0.007 | 0.100 | 0.124 | 0.025 | 0.005 | 0.026 | 0.005 | 0.010 |
|  | *0.018* | *0.066* | *0.005* | *0* | *0.173* | *0* | *0* | *0.002* | *0* | *0* |
| P3 | 0.010 | 0 | 0.005 | 0.100 | 0.460 | 0.025 | 0.005 | 0.030 | 0.005 | 0.010 |
|  | *0* | *0* | *0* | *0* | *0* | *0* | *0* | *0* | *0* | *0* |
| F1 | 0.025 | 0.005 | 0.005 | 0.100 | 0.110 | 0.025 | 0.005 | 0.025 | 0.005 | 0.010 |
|  | *0.021* | *0* | *0* | *0* | *0.042* | *0* | *0* | *0* | *0* | *0* |
| F2 | 0.010 | 0.005 | 0.006 | 0.100 | 0.168 | 0.025 | 0.005 | 0.029 | 0.005 | 0.010 |
|  | *0* | *0* | *0.003* | *0* | *0.259* | *0* | *0* | *0.008* | *0* | *0* |
| F3 | 0.040 | 0.007 | 0.005 | 0.100 | 0.186 | 0.025 | 0.005 | 0.025 | 0.005 | 0.010 |
|  | *0.037* | *0.003* | *0* | *0* | *0.097* | *0* | *0* | *0* | *0* | *0* |
| F5 | 0.016 | 0.005 | 0.012 | 0.100 | 0.069 | 0.025 | 0.014 | 0.065 | 0.005 | 0.010 |
|  | *0.018* | *0* | *0.014* | *0.000* | *0.063* | *0* | *0.016* | *0.118* | *0* | *0* |
| F6 | 0.017 | 0.183 | 0.007 | 0.100 | 0.287 | 0.025 | 0.005 | 0.053 | 0.006 | 0.010 |
|  | *0.021* | *0.241* | *0.005* | *0* | *0.263* | *0* | *0* | *0.075* | *0.002* | *0* |
| F8 | 0.017 | 0.005 | 0.006 | 0.162 | 0.277 | 0.025 | 0.005 | 0.138 | 0.005 | 0.010 |
|  | *0.021* | *0* | *0.002* | *0.198* | *0.687* | *0* | *0* | *0.359* | *0* | *0* |
| F10 | 0.018 | 0.005 | 0.005 | 0.100 | 0.373 | 0.025 | 0.005 | 0.029 | 0.005 | 0.010 |
|  | *0.020* | *0* | *0* | *0* | *0.798* | *0* | *0* | *0.010* | *0* | *0* |
| LOD (µg/L) | 0.02 | 0.01 | 0.01 | 0.2 | 0.02* | 0.05 | 0.01 | 0.05* | 0.01 | 0.02 |

*LOD of carbendazim and imidacloprid for samples collected in autumn 2012 was 0.01 µg/L

Chlor=chlorprofam, Pir-meth=pirimiphos-methyl, Tolc-meth=tolclophos-methyl, Carb=carbendazim, Ethiofen=ethiofencarb, Imidacl=imidacloprid, Ispr=isoproturon, Proch=prochloraz, Imaz=imazalil Meth=methiocarb, LOD=limit of detection

Table S3. Average and standard deviation (in italic) of macrofauna abundances on the level of Order at the sampling sites (from Ieromina et al, 2015)

|  | Hem | Dipt | Eph | Trich | Plec | Odo | Col | Lep | Meg | Col | Gast | Cypr | Rhyn | Hapl | Tricl | Isop | Ostr | Cycl | Dipl | Cop | Amp | Arg | Anos | Mys | Bas | Het | Ven | Neot | Arch | Aca |
| --- | --- | --- | --- | --- | --- | --- | --- | --- | --- | --- | --- | --- | --- | --- | --- | --- | --- | --- | --- | --- | --- | --- | --- | --- | --- | --- | --- | --- | --- | --- |
| D1 | 18.8 | 31.9 | 119.1 | 0.3 | 0.0 | 5.4 | 0.8 | 0.1 | 0.0 | 15.8 | 2.2 | 0.1 | 0.4 | 7.4 | 0.0 | 1.0 | 0.2 | 0.3 | 69.7 | 0.0 | 0.1 | 0.0 | 0.0 | 0.0 | 24.7 | 0.3 | 61.1 | 1.1 | 0.0 | 1.1 |
|  | *12.6* | *71.4* | *106.6* | *0.9* | *0.0* | *5.6* | *1.0* | *0.3* | *0.0* | *49.3* | *3.5* | *0.3* | *1.0* | *23.1* | *0.0* | *3.2* | *0.6* | *0.7* | *131.0* | *0.0* | *0.3* | *0.0* | *0.0* | *0.0* | *28.2* | *0.7* | *127.2* | *3.1* | *0.0* | *1.9* |
| D2 | 21.2 | 106.5 | 286.6 | 2.7 | 0.0 | 23.1 | 5.7 | 0.0 | 0.0 | 3.5 | 0.1 | 0.0 | 0.0 | 7.4 | 0.0 | 0.0 | 18.0 | 0.5 | 22.0 | 0.0 | 0.0 | 0.0 | 0.0 | 0.0 | 18.0 | 21.4 | 32.9 | 2.6 | 0.0 | 0.8 |
|  | *29.6* | *104.5* | *487.7* | *3.5* | *0.0* | *32.3* | *9.7* | *0.0* | *0.0* | *9.4* | *0.3* | *0.0* | *0.0* | *23.4* | *0.0* | *0.0* | *40.5* | *1.6* | *44.1* | *0.0* | *0.0* | *0.0* | *0.0* | *0.0* | *38.0* | *51.7* | *66.8* | *6.9* | *0.0* | *1.1* |
| D3 | 5.8 | 435.3 | 5.8 | 135.3 | 0.0 | 0.5 | 18.3 | 0.0 | 0.0 | 0.0 | 0.5 | 0.0 | 0.0 | 44.8 | 0.0 | 0.0 | 0.0 | 0.5 | 28.0 | 0.0 | 31.0 | 0.0 | 0.0 | 0.0 | 7.0 | 18.3 | 0.8 | 9.3 | 0.0 | 6.5 |
|  | *5.7* | *757.8* | *3.6* | *181.3* | *0.0* | *1.0* | *25.4* | *0.0* | *0.0* | *0.0* | *0.6* | *0.0* | *0.0* | *64.2* | *0.0* | *0.0* | *0.0* | *1.0* | *56.0* | *0.0* | *20.7* | *0.0* | *0.0* | *0.0* | *10.9* | *20.8* | *1.5* | *15.3* | *0.0* | *9.3* |
| D4 | 9.8 | 8.3 | 52.0 | 0.5 | 0.0 | 0.0 | 167.5 | 0.0 | 0.0 | 0.0 | 4.0 | 0.0 | 0.0 | 38.5 | 0.0 | 12.5 | 0.0 | 0.5 | 324.3 | 0.0 | 4.5 | 0.0 | 0.0 | 0.0 | 7.0 | 8.3 | 0.0 | 0.3 | 0.0 | 0.0 |
|  | *10.7* | *6.3* | *31.1* | *0.6* | *0.0* | *0.0* | *333.7* | *0.0* | *0.0* | *0.0* | *8.0* | *0.0* | *0.0* | *51.9* | *0.0* | *13.0* | *0.0* | *1.0* | *273.5* | *0.0* | *4.2* | *0.0* | *0.0* | *0.0* | *2.9* | *15.8* | *0.0* | *0.5* | *0.0* | *0.0* |
| P1 | 44.1 | 7.5 | 39.1 | 0.6 | 0.2 | 0.8 | 2.2 | 0.0 | 0.3 | 0.9 | 6.3 | 0.1 | 0.9 | 45.8 | 0.0 | 2.1 | 2.0 | 3.2 | 223.7 | 7.8 | 0.1 | 0.6 | 0.0 | 0.9 | 27.9 | 24.7 | 9.9 | 6.3 | 0.1 | 1.0 |
|  | *56.8* | *8.4* | *34.4* | *1.1* | *0.6* | *1.3* | *3.8* | *0.0* | *0.9* | *1.5* | *9.3* | *0.3* | *1.1* | *98.6* | *0.0* | *3.1* | *6.3* | *5.5* | *455.1* | *16.8* | *0.3* | *1.1* | *0.0* | *1.4* | *22.4* | *28.8* | *24.4* | *10.9* | *0.3* | *1.6* |
| P2 | 37.8 | 4.5 | 45.1 | 0.4 | 0.4 | 0.5 | 1.0 | 0.0 | 1.7 | 1.9 | 1.2 | 0.0 | 1.0 | 13.4 | 0.2 | 2.9 | 0.0 | 2.1 | 1032.7 | 86.5 | 0.6 | 0.1 | 0.0 | 1.0 | 35.7 | 17.5 | 5.1 | 4.6 | 0.0 | 3.4 |
|  | *41.3* | *9.2* | *69.5* | *0.7* | *1.3* | *0.8* | *1.5* | *0.0* | *5.0* | *5.0* | *2.8* | *0.0* | *1.6* | *15.8* | *0.6* | *4.6* | *0.0* | *2.8* | *3241.5* | *271.8* | *1.9* | *0.3* | *0.0* | *1.8* | *52.6* | *16.9* | *5.2* | *4.8* | *0.0* | *8.0* |
| P3 | 20.5 | 3.5 | 132.5 | 1.3 | 0.0 | 5.0 | 2.5 | 1.0 | 0.3 | 0.3 | 1.0 | 1.0 | 0.3 | 17.5 | 0.0 | 5.8 | 0.0 | 5.0 | 290.3 | 0.0 | 5.5 | 0.0 | 0.0 | 0.0 | 10.5 | 7.8 | 1.0 | 8.3 | 0.0 | 2.8 |
|  | *19.1* | *2.6* | *80.8* | *1.9* | *0.0* | *5.0* | *3.3* | *2.0* | *0.5* | *0.5* | *1.4* | *1.4* | *0.5* | *12.6* | *0.0* | *5.1* | *0.0* | *7.1* | *274.8* | *0.0* | *6.4* | *0.0* | *0.0* | *0.0* | *6.6* | *12.3* | *1.4* | *7.4* | *0.0* | *5.5* |
| P4 | 11.5 | 1.5 | 34.0 | 0.0 | 0.0 | 0.5 | 3.0 | 0.8 | 0.0 | 0.8 | 0.5 | 0.0 | 2.0 | 50.0 | 0.0 | 12.8 | 0.0 | 0.3 | 104.8 | 0.0 | 2.0 | 0.0 | 0.0 | 0.0 | 47.3 | 9.3 | 3.8 | 8.5 | 0.0 | 0.5 |
|  | *11.1* | *0.6* | *44.0* | *0.0* | *0.0* | *1.0* | *4.8* | *1.5* | *0.0* | *1.0* | *1.0* | *0.0* | *2.3* | *57.7* | *0.0* | *5.4* | *0.0* | *0.5* | *196.9* | *0.0* | *2.2* | *0.0* | *0.0* | *0.0* | *41.7* | *15.2* | *4.1* | *7.7* | *0.0* | *0.6* |
| F1 | 49.0 | 6.2 | 12.8 | 0.1 | 0.1 | 0.3 | 1.2 | 0.0 | 0.0 | 0.1 | 2.1 | 0.7 | 0.0 | 24.2 | 0.4 | 2.0 | 0.0 | 6.5 | 155.1 | 1.0 | 1.8 | 0.1 | 0.3 | 5.5 | 12.5 | 24.5 | 5.3 | 2.4 | 0.0 | 4.3 |
|  | *48.2* | *12.3* | *12.3* | *0.3* | *0.3* | *0.7* | *1.4* | *0.0* | *0.0* | *0.3* | *6.0* | *1.3* | *0.0* | *26.7* | *1.3* | *2.3* | *0.0* | *9.3* | *311.6* | *3.2* | *2.9* | *0.3* | *0.9* | *10.9* | *18.6* | *23.4* | *9.7* | *3.0* | *0.0* | *6.0* |
| F2 | 27.5 | 3.3 | 18.6 | 1.2 | 0.0 | 0.0 | 1.3 | 0.1 | 0.2 | 0.1 | 6.3 | 0.0 | 0.9 | 13.1 | 0.0 | 1.7 | 0.0 | 0.6 | 25.3 | 1.2 | 0.3 | 0.0 | 0.0 | 2.4 | 63.1 | 47.8 | 10.1 | 31.3 | 0.0 | 0.2 |
|  | *63.0* | *8.1* | *32.0* | *3.5* | *0.0* | *0.0* | *3.8* | *0.3* | *0.4* | *0.3* | *13.0* | *0.0* | *1.4* | *20.2* | *0.0* | *1.7* | *0.0* | *1.3* | *47.7* | *3.8* | *0.5* | *0.0* | *0.0* | *4.1* | *49.3* | *57.4* | *11.1* | *27.9* | *0.0* | *0.4* |
| F3 | 1.7 | 39.5 | 7.5 | 0.3 | 0.0 | 0.0 | 2.2 | 0.0 | 0.0 | 0.0 | 0.7 | 0.0 | 0.7 | 30.7 | 0.3 | 2.0 | 0.0 | 10.0 | 6.5 | 0.0 | 0.2 | 0.0 | 0.0 | 0.8 | 19.2 | 17.0 | 1.5 | 2.2 | 0.0 | 0.8 |
|  | *2.3* | *59.3* | *8.8* | *0.8* | *0.0* | *0.0* | *3.3* | *0.0* | *0.0* | *0.0* | *1.2* | *0.0* | *1.0* | *32.0* | *0.8* | *2.1* | *0.0* | *15.8* | *9.5* | *0.0* | *0.4* | *0.0* | *0.0* | *1.3* | *23.0* | *19.9* | *2.8* | *2.7* | *0.0* | *2.0* |
| F4 | 19.5 | 23.1 | 21.7 | 0.0 | 0.0 | 0.1 | 0.9 | 0.0 | 1.6 | 1.7 | 3.8 | 0.1 | 4.4 | 10.4 | 1.3 | 2.3 | 0.0 | 46.1 | 1197.5 | 0.0 | 0.4 | 0.3 | 0.0 | 0.4 | 32.4 | 38.7 | 4.3 | 2.0 | 0.0 | 0.0 |
|  | *27.4* | *65.1* | *28.3* | *0.0* | *0.0* | *0.3* | *1.1* | *0.0* | *4.7* | *4.7* | *4.0* | *0.3* | *6.0* | *15.5* | *4.1* | *4.6* | *0.0* | *62.4* | *1684.6* | *0.0* | *1.3* | *0.9* | *0.0* | *1.0* | *24.8* | *29.1* | *6.4* | *3.9* | *0.0* | *0.0* |
| F5 | 16.0 | 1.4 | 26.8 | 0.0 | 0.0 | 0.0 | 0.8 | 0.0 | 0.0 | 0.0 | 9.4 | 0.0 | 0.7 | 69.5 | 0.1 | 2.6 | 0.0 | 78.7 | 782.0 | 0.0 | 0.2 | 0.0 | 0.0 | 0.3 | 38.8 | 83.8 | 3.5 | 5.2 | 1.2 | 0.7 |
|  | *17.5* | *2.0* | *33.1* | *0.0* | *0.0* | *0.0* | *1.1* | *0.0* | *0.0* | *0.0* | *27.6* | *0.0* | *1.1* | *83.1* | *0.3* | *2.6* | *0.0* | *225.7* | *1321.4* | *0.0* | *0.4* | *0.0* | *0.0* | *0.7* | *36.2* | *96.1* | *6.4* | *6.3* | *3.8* | *1.3* |
| F6 | 47.8 | 6.9 | 7.5 | 1.7 | 0.0 | 0.0 | 2.4 | 0.0 | 0.1 | 3.6 | 2.6 | 0.1 | 1.2 | 38.6 | 0.5 | 3.5 | 0.0 | 7.0 | 60.1 | 0.3 | 0.0 | 0.6 | 0.0 | 0.1 | 50.9 | 17.4 | 0.4 | 0.4 | 0.0 | 0.6 |
|  | *63.3* | *12.9* | *8.5* | *3.9* | *0.0* | *0.0* | *3.4* | *0.0* | *0.3* | *11.4* | *3.7* | *0.3* | *2.1* | *55.4* | *1.6* | *10.0* | *0.0* | *15.0* | *117.6* | *0.9* | *0.0* | *1.3* | *0.0* | *0.3* | *60.7* | *18.7* | *0.7* | *0.7* | *0.0* | *1.1* |
| F7 | 33.2 | 26.7 | 58.8 | 0.3 | 0.0 | 0.3 | 0.7 | 0.0 | 0.0 | 1.7 | 33.3 | 0.0 | 0.3 | 128.6 | 0.1 | 3.0 | 0.0 | 8.5 | 331.7 | 0.3 | 0.0 | 0.0 | 0.1 | 0.1 | 98.8 | 20.1 | 5.7 | 4.2 | 0.0 | 0.1 |
|  | *48.8* | *62.3* | *53.6* | *0.9* | *0.0* | *0.9* | *0.9* | *0.0* | *0.0* | *5.4* | *70.1* | *0.0* | *0.5* | *240.2* | *0.3* | *7.2* | *0.0* | *21.8* | *629.9* | *0.9* | *0.0* | *0.0* | *0.3* | *0.3* | *212.5* | *16.0* | *11.3* | *5.7* | *0.0* | *0.3* |
| F8 | 32.2 | 4.5 | 31.0 | 0.1 | 0.0 | 0.1 | 1.6 | 0.0 | 0.2 | 1.0 | 12.3 | 1.3 | 0.7 | 11.0 | 0.0 | 2.5 | 0.0 | 7.9 | 19.5 | 4.5 | 0.4 | 0.0 | 0.0 | 0.9 | 77.0 | 25.5 | 1.9 | 8.7 | 0.0 | 1.5 |
|  | *28.5* | *5.8* | *28.9* | *0.3* | *0.0* | *0.3* | *2.9* | *0.0* | *0.6* | *3.2* | *22.2* | *4.1* | *0.7* | *11.9* | *0.0* | *4.2* | *0.0* | *12.4* | *42.8* | *14.2* | *1.3* | *0.0* | *0.0* | *2.8* | *66.4* | *27.4* | *2.4* | *10.8* | *0.0* | *3.2* |
| F9 | 51.2 | 5.3 | 194.3 | 0.5 | 0.0 | 1.0 | 0.0 | 0.0 | 0.5 | 0.0 | 2.8 | 0.5 | 3.3 | 11.8 | 0.0 | 32.5 | 0.0 | 77.3 | 1345.2 | 10.8 | 3.7 | 0.0 | 0.0 | 0.0 | 50.8 | 22.7 | 6.5 | 9.5 | 0.0 | 2.7 |
|  | *36.4* | *5.1* | *250.8* | *0.8* | *0.0* | *2.4* | *0.0* | *0.0* | *0.8* | *0.0* | *3.7* | *1.2* | *5.4* | *21.8* | *0.0* | *47.7* | *0.0* | *106.8* | *1452.2* | *16.8* | *5.4* | *0.0* | *0.0* | *0.0* | *34.3* | *22.2* | *4.2* | *17.0* | *0.0* | *3.4* |
| F10 | 19.1 | 2.4 | 30.2 | 0.1 | 0.0 | 0.1 | 1.9 | 0.0 | 0.1 | 0.0 | 4.2 | 0.1 | 1.2 | 6.8 | 0.3 | 8.3 | 0.0 | 10.4 | 2155.0 | 0.0 | 0.6 | 0.0 | 0.0 | 0.0 | 130.6 | 70.1 | 2.0 | 35.2 | 0.0 | 1.5 |
|  | *12.4* | *3.9* | *38.2* | *0.3* | *0.0* | *0.3* | *3.2* | *0.0* | *0.3* | *0.0* | *8.8* | *0.3* | *2.6* | *13.2* | *0.9* | *9.6* | *0.0* | *22.6* | *4252.7* | *0.0* | *1.3* | *0.0* | *0.0* | *0.0* | *149.5* | *84.5* | *2.3* | *43.9* | *0.0* | *2.9* |

Hem = Hemiptera, Dipt = Diptera, Eph = Ephemeroptera, Trich = Trichoptera, Plec = Plecoptera, Odo = Odonata, Col = Coleoptera, Lep = Lepidoptera, Meg = Megaloptera, Colm = Collembola, Gast = Gasterosteiformes, Cypr = Cypriniformes, Rhyn = Rhynchobdellida, Hapl = Haplotaxida, Tricl = Tricladida, Isop = Isopoda, Ostr = Ostracoda, Cycl = Cyclopoida, Dipl = Diplostraca, Cop = Copepoda, Amp = Amphipoda, Arg = Arguloida, Anos = Anostraca, Mys = Mysida, Bas = Basommatophora, Het = Heterostropha, Ven = Veneroida, Neot = Neotaenioglossa, Arch = Architaenioglossa, Aca = Acari, Tricl = Tricladida.

TableS 4. Variance inflation factors (VIF) for explanatory variables

| Explanatory variable | VIF |
| --- | --- |
| Tepmerature | 3.07 |
| Dissolved oxygen | 1.37 |
| Dissolved organic carbon | 1.70 |
| P04- | 1.59 |
| Macrophyte coverage | 1.37 |
| NO2- | 4.01 |
| NO3- | 4.17 |
| Chloorprofam | 1.54 |
| Pirimifos-methyl | 1.45 |
| Tolclofos-methyl | 1.07 |
| Prochloraz | 1.32 |
| Carbendazim | 1.29 |
| Imazalil | 1.41 |
| Imidacloprid | 1.20 |
| Isoproturon | 1.11 |

Fig. S1. Schematic map of the research area: all sites sampled (names of the sampling sites underlined) (Fig. 1A), and detailed map of the sites sampled in area 1 highlighted in Fig 1A (Fig. 1B) inculiding the hydrological feautes in the area (from Ieromina et al, 2015)

| 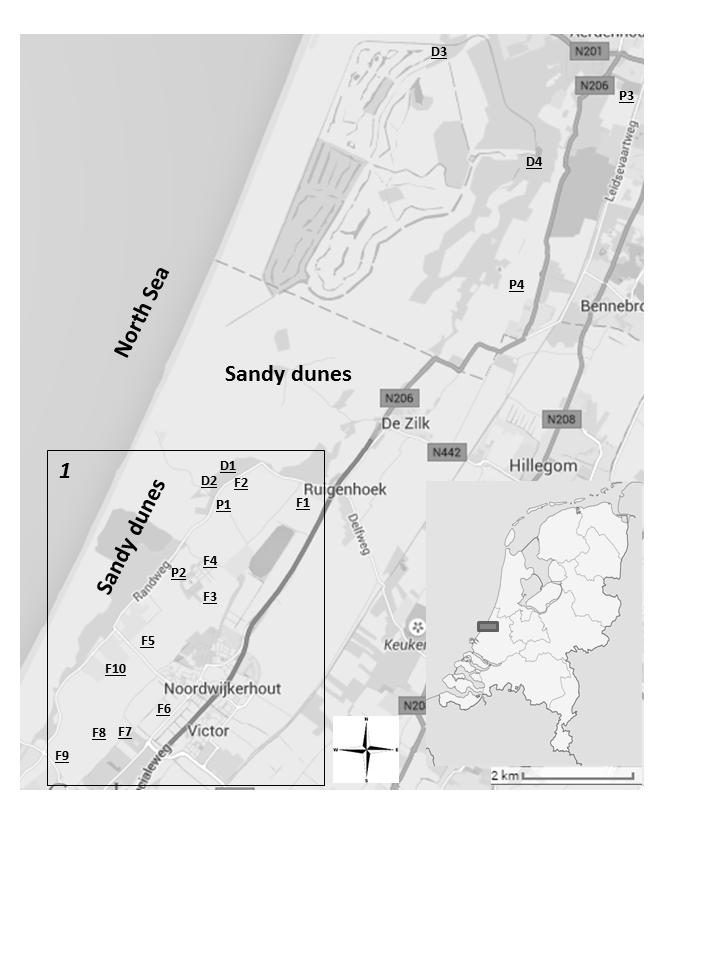  **A** | 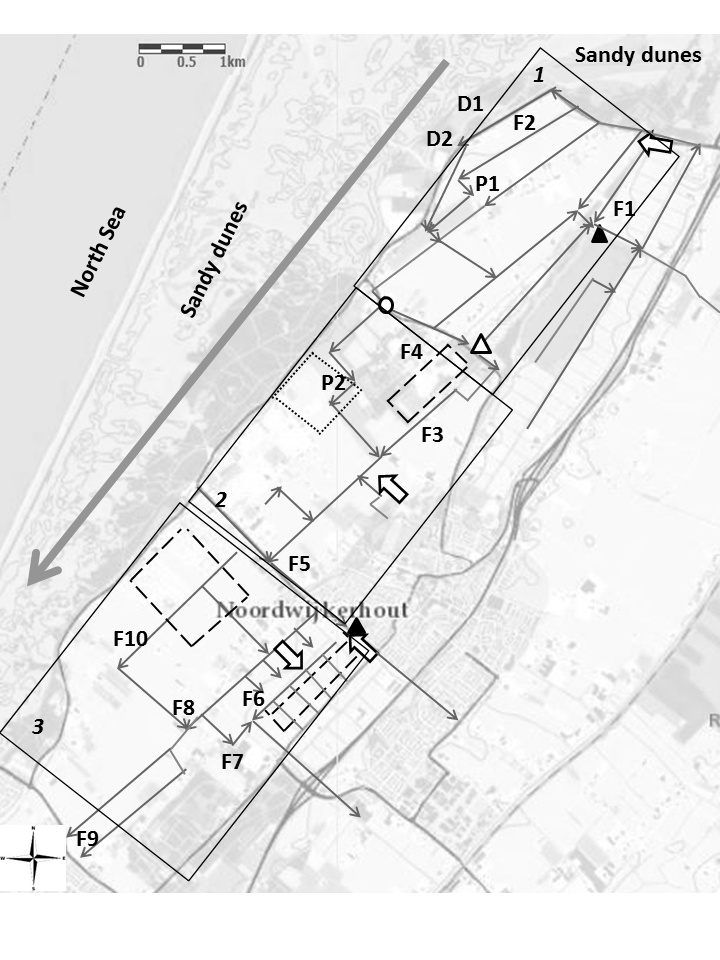  **B** |
| --- | --- |

| 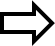 | Inlet | 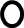 | Inlet to the other polder | |
| --- | --- | --- | --- | --- |
| 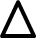 | Emergency outlet |  | Low water area |  |
|  | Direction of the water flow |  | High water area |  |
| 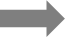 | Direction of the elevation gradient |  | Line separating polders |  |
| 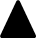 | Regular outlet |  |  |  |

1 – Polder Het Langeveld (polder 1)

2 –Noordzijderpolder-Noord (polder 2 North)

3 – Noordzijderpolder-Zuid (polder 2 South)

D- sites in the watersheds in the nature reserve

F- sites in the ditches next to flower fields

P-sites in the ditches next to the pastures
